# Tad Pili Play a Dynamic Role in Caulobacter crescentus Surface Colonization

**DOI:** 10.1128/mBio.01237-19

**Published:** 2019-06-18

**Authors:** Matteo Sangermani, Isabelle Hug, Nora Sauter, Thomas Pfohl, Urs Jenal

**Affiliations:** a Biozentrum, University of Basel, Basel, Switzerland; b Department of Chemistry, University of Basel, Basel, Switzerland; c Swiss Nanoscience Institute, Basel, Switzerland; d Institute of Physics, University of Freiburg, Freiburg, Germany; Max Planck Institute for Terrestrial Microbiology; University of Nottingham; University of California at Santa Cruz

**Keywords:** *Caulobacter*, type IV pili, c-di-GMP, surface sensing

## Abstract

Bacterial surface attachment is mediated by filamentous appendages called pili. Here, we describe the role of Tad pili during surface colonization of Caulobacter crescentus. Using an optical trap and microfluidic controlled flow conditions to mimic natural environments, we demonstrated that Tad pili undergo repeated dynamic cycles of extension and retraction. Within seconds after establishing surface contact, pilus retraction reorients cells into an upright position, promoting walking-like movements against the medium flow. Pilus-mediated positioning of the flagellate pole close to the surface facilitates motor-mediated mechanical sensing and promotes anchoring of the holdfast, an adhesive substance that affords long-term attachment. We present evidence that the second messenger c-di-GMP regulates pilus dynamics during surface encounter in distinct ways, promoting increased activity at intermediate levels and retraction of pili at peak concentrations. We propose a model in which flagellum and Tad pili functionally interact and together impose a ratchet-like mechanism that progressively drives C. crescentus cells toward permanent surface attachment.

## Abstract (precis)

Bacteria are able to colonize surfaces in environmental, industrial, and medical settings, where they form resilient communities called biofilms. In order to control bacterial surface colonization, microbiologists need to gain a detailed understanding of the processes that bacteria use to live at the liquid-surface interface and that allow them to adhere to and move on surfaces and eventually grow and persist on solid media. To facilitate these processes, bacteria are equipped with adhesive structures such as flagella and pili and with matrix components such as exopolysaccharides. How these cellular organelles are coordinated to optimize surface processes is currently subject to intense investigations. Here we used the model organism Caulobacter crescentus to demonstrate that polar pili are highly dynamic structures that are functionally interconnected with the flagellar motor to mediate surface sensing, thereby enforcing rapid and permanent surface attachment. These studies provide an entry point for an in-depth molecular analysis of bacterial surface colonization.

## INTRODUCTION

Bacteria have evolved effective mechanisms to colonize abiotic and biotic surfaces in order to scavenge nutrients, attack host tissue, or assemble into resilient communities called biofilms. A pivotal role in this process is played by adhesive pili, also called fimbriae, which are protein-based filaments exposed on the surface of bacteria that have adopted different functions, including adherence, motility, electron transfer, acquisition of DNA, and protein secretion (1, 2). Accordingly, pili are crucial virulence factors during infection processes (3). They mediate direct contact between pathogens and specific host tissues and promote pathogen spreading and cellular invasion (4–7). The highly corrugated surface of the extended pilus filaments mediates attachment to hydrophobic and hydrophilic surfaces through reversible, nonspecific interactions (8, 9). Type IV pili represent the most sophisticated class of these filaments. The members of the best-studied subgroup, type IVa, are dynamic machineries that undergo cycles of extension and retraction through the rapid assembly and disassembly of pilin subunits at the proximal end of the structure (6, 10). Extension and retraction are powered by specific cytoplasmic ATPases, which generate rotational movements of the assembly platform in the inner membrane to incorporate pilin subunits into or extract them from the helical filaments (4, 11). Through the coordinated extension and retraction of multiple polar pili, single cells are able to move on surfaces and explore their environments (1, 12, 13). Two types of pilus-mediated movements have been described (14). Crawling movements of horizontally positioned cells, called twitching, can result in traversal of large distances with high directional persistence (15–17). In contrast, walking movements of orthogonal upright cells facilitate rapid exploration of smaller areas (18).

**FIG 1:**
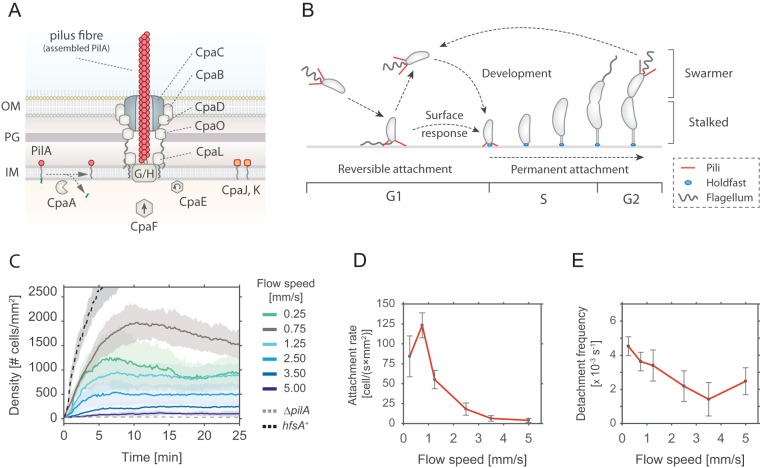
Pilus-mediated surface attachment of C. crescentus cells under flow conditions. (A) Schematic model of the Tad pilus machinery in C. crescentus. The function of individual components is drawn according to results of comparative analysis of the *cpa* locus with other Tad pili (19, 22). PilA is the major pilin subunit that matures upon removal of the signal peptide by the prepilin peptidase CpaA and is then assembled into a filament by the inner membrane platform that is located at the base of the filament (CpaG and CpaH). The CpaF ATPase is the functional motor protein, while the CpaE ATPase is required for polar localization of the pilus machinery (19, 22). Minor pilin subunits (CpaJ and CpaK) and envelope-spanning components of the pilus machinery are indicated. OM, outer membrane; PG, peptidoglycan; IM, inner membrane. (B) Schematic of the C. crescentus cell cycle. SW cells are born with assembled pili (red) and flagellum (gray). Upon surface encounter of the SW cell, pili promote temporary attachment and position the flagellate pole close to the surface. This triggers the secretion of an adhesive exopolysaccharide, the holdfast (blue), and results in permanent attachment of the cell. Attached cells differentiate into ST cells, initiating the division cycle that generates another motile SW cell. (C) The velocity of the fluid flow influences pilus-mediated surface attachment. The chart shows numbers of C. crescentus cells that lacked an adhesive holdfast (NA1000) adhering to the flow channel surface at different flow velocities. Results from experiments performed with a wild-type strain able to produce holdfast (*hfsA^+^*) and with a mutant lacking pili (Δ*pilA*) are shown as controls at a flow speed of 0.75 mm/s. Opaque areas represent standard deviations (*n *>* *3). (D) Average number of newly attached SW cells per square millimeter per second at different flow velocities. The values represent averages of results from the microfluidic attachment assay represented in panel C and were scored between 10 and 25 min. Error bars represent standard deviations (*n *>* *3). (E) Frequency of surface detachment at different flow velocities. Detachment frequencies represent the ratio between the number of cells leaving the surface and the total number attached. The values represent averages of results from the microfluidic attachment assay represented in panel C scored between 10 and 25 min. Error bars represent standard deviations (*n *>* *3).

Type IV pili are widespread in bacteria and archaea (11, 12). Distinctive features divide these structures into two classes (type IVa and type IVb). Type IVa represents a uniform class that is found in important human pathogens such as Pseudomonas aeruginosa, Vibrio cholerae, and *Neisseria* spp. and in environmental bacteria such as Myxococcus xanthus, Shewanella putrefaciens, and Bdellovibrio bacteriovorus. The type IVb subclass is less homogenous and is best characterized for enteropathogenic Escherichia coli or V. cholerae (19). A subclass of type IVb pili is represented by the tight adherence (Tad) or Flp (fimbrial low-molecular-weight protein) pili (Fig. 1A) (20–22). Sometimes classified as a separate type IVc group, Tad pilin subunits are smaller than the pilins of other type IV systems but show similar hydrophobic intermolecular interactions providing the main force holding the fibers together (13, 19, 21). Tad pili promote surface colonization, cell-to-cell aggregation, and biofilm cohesion and promote virulence of different bacterial pathogens (13, 19, 21, 23–25). In contrast to type IVa and other type IVb pilus systems, Tad clusters seem to lack a dedicated retraction ATPase and most Tad pili do not seem to be dynamic (19). However, recent studies indicated that toxin-coregulated pili (TCP) of V. cholerae and Tad pili of C. crescentus are retractable (26, 27). It was proposed for TCP that incorporation of minor pilin subunits (TcpB) into a growing pilus can block assembly and trigger a spontaneous disassembly and retraction event.

In C. crescentus, polar Tad pili facilitate the attachment of planktonic cells to surfaces (20, 27, 28). During C. crescentus division, a polarized sessile stalked (ST) cell produces a motile offspring, the swarmer (SW) cell, which is equipped with a single flagellar propeller and multiple pili (Fig. 1B) (20, 29). The newborn SW cell remains in a motile, nonreplicating state for a defined period called G_1_. After this period, chromosome replication resumes coincident with cell differentiation, during which flagellum and pili are replaced by an adhesive exopolysaccharide (EPS), the holdfast, and the stalk. While the developmental program defines an extended time window of motility, SW cells that are challenged with surface are able to transit to the sessile state within seconds (28, 30, 31). This process is executed by a surface recognition program that involves sensing of mechanical cues and triggering of a burst of c-di-GMP, a second messenger that controls the motile-sessile transition in a wide range of bacteria (32–34). In turn, c-di-GMP allosterically activates a preassembled holdfast synthesis machinery to irreversibly anchor cells that encounter a surface (28, 35).

Although the initial surface contact and adherence are indisputably mediated by pili, different views have been put forward for the role of polar Tad pili in C. crescentus surface sensing. In one model, dynamic action of pili positions the flagellar pole in close contact with the surface to allow mechanosensation by the membrane-integral rotary motor, triggering a spike of c-di-GMP through the activation of a motor-associated diguanylate cyclase (28). A second model proposed that surface-bound Tad pili themselves serve as surface sensors, mediating an internal upshift of c-di-GMP levels after experiencing resistance upon retracting (27). In line with the first model, recent studies demonstrated that flagellar motors, but not type IVa pili, are required for surface sensing in P. aeruginosa (36) and that a flagellar motor-coupled diguanylate cyclase increases levels of c-di-GMP in this organism (37). Moreover, studies in V. cholerae and P. aeruginosa had shown that c-di-GMP is positioned upstream of type IV pili and that this second messenger regulates pilus assembly and activity (36, 38–41). In line with the second model, type IV pili were recently shown to be required for a surface-mediated increase of cAMP and virulence gene expression in P. aeruginosa (42).

To more closely scrutinize the role of Tad pili in C. crescentus surface recognition and surface colonization, we carefully analyzed their dynamic behavior and regulation. We used microfluidics to perform experiments under controlled flow conditions. We demonstrate that under conditions of steady medium flow, Tad-mediated cell attachment is transient, offering motile bacteria a short window of opportunity during which they can sense a surface and trigger holdfast biogenesis. Tad pili are already highly dynamic before the motile SW cell separates from its ST mother, explaining the observed ultrarapid surface recognition capability of newborn SW cells (28). We show that Tad pili can go through multiple rounds of extension and retraction, mediating walking-like motility on surfaces. Finally, we present data indicating that the flagellar motor and Tad pili functionally interact and that an increase in the c-di-GMP concentration results in retraction of pili. Together, these data led to a proposal of a model in which highly dynamic Tad pilus structures are integrated as part of a complex mechanism that senses mechanical stimuli upon C. crescentus surface encounter to promote and accelerate surface anchoring.

## RESULTS

### Tad pili mediate transient surface attachment under conditions of medium flow.

Surface adhesion of C. crescentus via its polar pili is transient and weaker than the strong and long-lasting attachment via the adhesive holdfast (43). To investigate the overall contribution of pili to surface attachment without interference of the holdfast, we analyzed the behavior of a C. crescentus holdfast mutant (NA1000) (44) and scored attachment efficiency in simple microfluidic channels with a single inlet supplying a culture with a constant flow of medium. Microscopy time-lapse images were recorded to determine the rate of surface attachment. Importantly, mutants lacking pili were unable to adhere to the glass surface in such an assay (Fig. 1C).

The number of attached cells scaled with the flow velocity of the medium (Fig. 1C). We observed a plateau in the density of attached cells per unit surface area, arguing that the attachment was transient and that, under these conditions, attachment and detachment of bacteria reached an equilibrium. A plateau was generally reached 5 to 10 min after the start of the experiment. Colonization density at equilibrium showed a strong dependency on the flow velocity. The highest density of attached cells was observed with a fluid flow velocity in the microchannel of 0.75 mm/s (maximal velocity in the middle of the channel). This velocity was chosen as the standard for further experiments. At lower flow speed, we measured a lower plateau value of colonization density (Fig. 1C) due to a decreased rate of attachment of cells, while the detachment frequency was unchanged compared to the optimal flow velocity of 0.75 mm/s (Fig. 1D and E). Similarly, with increasing flow velocities, colonization densities also decreased, with attachment being completely abolished above 5 mm/s/cell (Fig. 1C). The decreased plateau levels were primarily due to a 2-fold-to-10-fold decrease in attachment rates, while the detachment frequencies were 2-fold-to-3-fold lower, meaning that the attached cells were less likely to leave (Fig. 1D and E). As a consequence, the average residence time of piliated SW cells on the glass surface was increased at higher flow (see Fig. S1A in the supplemental material). At a flow rate of 0.75 mm/s, 30% of the cells were retained on the surface for more than 2 min, whereas the fraction increased to 50% to 60% at higher flow rates.

10.1128/mBio.01237-19.1FIG S1(A) Residence time of cells on a surface during pilus-mediated attachment. Each curve indicates the cumulative fraction of cells residing on a surface for a period equal to or greater than the indicated time. The C. crescentus holdfast mutant (NA1000) used in these experiments was tested at different flow velocities. Opaque areas represent standard deviations (*n *>* *3). (B) Theoretical drag force experienced by a typical SW cell (red) and ST cell (gray) attached to a surface at different flow rates. The drag force was calculated using the equation shown below, and the bacterial shape was simplified as a sphere near a surface with a volume equivalent to that of the average cell. The drag was calculated assuming a distance from the surface (*h*) of 0.25 μm.
$$F_{\text{Drag}}=\frac{3\text{π }a\text{ μ }u_{z}}{2\;(\;-\;\frac{0.0625\;a^{5}}{h^{5}}\;-\;\frac{0.1758\;a^{4}}{h^{4}}\;+\;\frac{0.125\;a^{3}}{h^{3}}\;-\;\frac{0.5625\;a}{h}+1)}$$
where *a* = radius of the sphere with volume equivalent to that of a cell (0.62 μm^3^ for SW cells and 0.71 μm^3^ for ST cells), *h* = height from the surface, μ = viscosity, and *u_z_* = flow velocity at height *h* from the surface (see equation 7.4.37 in reference 57). Download FIG S1, PDF file, 1.1 MB.Copyright © 2019 Sangermani et al.2019Sangermani et al.This content is distributed under the terms of the Creative Commons Attribution 4.0 International license.

Surface-bound cells subjected to fluid flow experience a drag proportional to the flow velocities (Fig. S1C). The observed increase in residence time at higher flow velocities may result from conformational changes in surface-attached pilus fibers that strengthen the interaction with the surface at higher drag forces (45, 46). Together, these observations demonstrated that pili mediate transient surface attachment of C. crescentus SW cells under conditions of medium flow. Thus, while pilus-mediated surface adherence provides a short time window for tactile sensing and holdfast production, a strain lacking holdfast machinery is unable to attach permanently (28). In contrast, cells harboring intact holdfast machinery (*hfsA*^+^) rapidly attach to and permanently colonize glass surfaces (Fig. 1C).

### Tad pili are dynamic and active before cell division.

**FIG 2:**
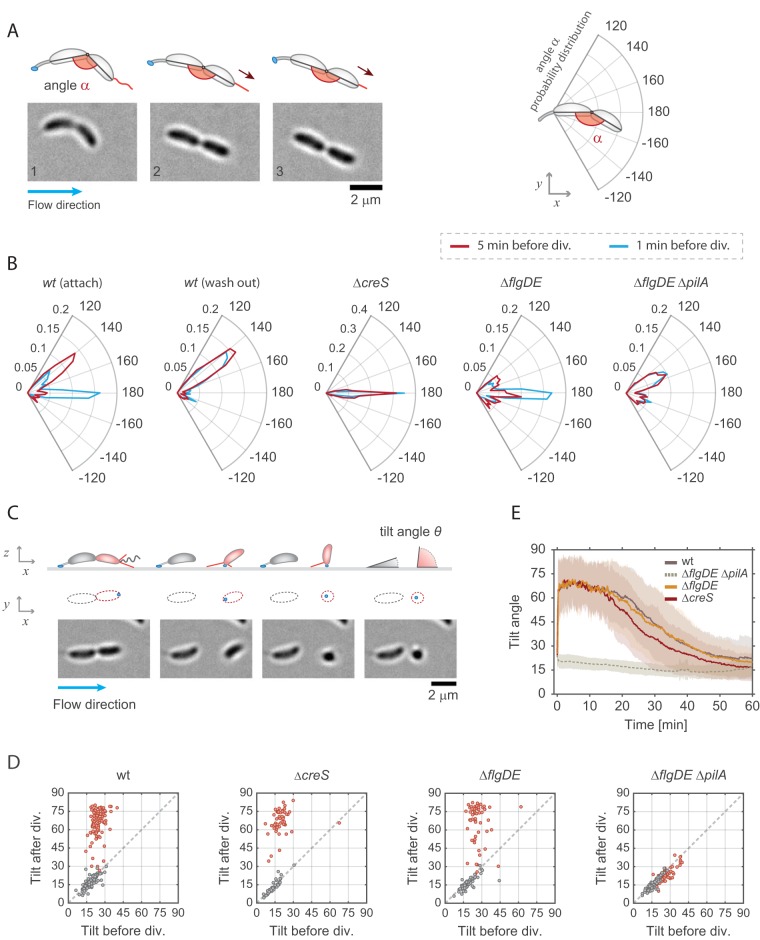
Pili are active before cell separation and position surface-bound cells upright afterward. (A) Image sequence of a crescentoid predivisional cell that is attached via its holdfast at one pole and is stretching due to the activity of pili located at the opposite pole. A schematic representation of the cell with its holdfast (blue) and pili (red) is shown above the micrographs, illustrating how angle α was determined in each experiment. The direction of medium flow is indicated. (B) Angle distribution along the main axis of predivisional cells. A schematic of a predivisional cell with the angles determined for ST and SW progeny is shown on the top right of the polar charts. Each plot shows the distribution of angle *α* in a different strain recorded 5 min (dark red) and 1 min (blue) before cell division. The C. crescentus wild-type strain had a peak at about 150° resulting from the crescentoid shape of predivisional cells at rest. A second peak was observed at 180° resulting from pilus retraction and the cell stretching into a straight line. Controls lacking pili (Δ*pilA*), crescentin (Δ*creS*), or the flagellum (Δ*flgDE*) are shown. Stretching of dividing cells that retained the SW offspring on the surface after separation (“attach”) and cell that failed to attach (“wash out”) are indicated. Number of replicates: wt (attach) = 130, wt (wash out) = 119, Δ*creS* = 56, Δ*flgDE* = 57, Δ*flgDE* Δ*pilA* = 75. (C) Time-lapse images of C. crescentus cell division under flow conditions (bottom) and a schematic view (top). A ST mother cell attached to the surface via its holdfast (blue) produces a SW offspring (red). A schematic representation of the cell outline in the *xy* plane is shown as identified by the analysis program. Polar pili are highlighted (red). Newborn SW cells but not ST mothers were able to move into a vertical position after separation. The tilt angles (θ, representing the angle between the main cell bod*y* axis and the glass surface) were calculated from the cell contour shape (*xy* plane), with the cells lying parallel to the glass surface and the cells in an upright position scoring θ = 0° and θ = 90°, respectively. The direction of medium flow is indicated. (D) Pili are required for newborn SW cells to move into an upright position. Scatter plots comparing the average angle *θ* of the same cells recorded 5 min before and 5 min after cell division are shown. The results shown were obtained with the strains indicated. Data for SW cells are in red, and data for ST cells are in gray. (E) Dynamics of pilus-mediated standing up of SW cells. The change of the angle *θ* after the birth (time point 0) of SW cells is shown over time for the C. crescentus wild-type strain and Δ*creS* and Δ*flgDE* Δ*pilA* mutants. Please note that the Δ*pilA* control strain also contained a Δ*flgDE* deletion, as cells lacking the external parts of the flagellum show hypersensitive surface response (28) and thus allowed trapping of enough pilus mutant cells on surfaces for this analysis. Solid lines represent average values; opaque areas show standard deviations. Number of replicates in panels D and E: wt = 98; strain Δ*flgDE* = 57; strain Δ*creS* = 56; strain Δ*flgDE* Δ*pilA* = 75.

Surface-attached C. crescentus SW cells were most often found standing upright in an orientation perpendicular to the surface, irrespective of their ability to synthesize an adhesive holdfast (28). This is an unfavorable position, considering the constant drag force from the medium flow. Also, after landing, cells occasionally moved a few microns against the medium flow in an upright position. Since only an active force could move cells against or position them in an orientation perpendicular to the flow, we speculated that surface-bound Tad pili are able to retract under these conditions (18). To investigate pilus dynamics, we first analyzed strains that are capable of secreting an adhesive holdfast in flow channels mimicking conditions that C. crescentus encounters in its natural environment (31). Under such conditions, offspring of attached dividing mothers are exposed to surface before division as a consequence of medium flow over the crescentoid dividing cells (Fig. 2) (31).

To investigate if pili are already able to retract before cells divide, movements of surface-attached dividing cells were carefully analyzed. We observed that the piliated pole of late predivisional cells was pulled away from the ST pole, thereby stretching the typical crescentoid cell shape into a straight line (Fig. 2A). To quantify this behavior, we determined the angle (*α*) between the two cell bodies 5 min and 1 min prior to cell separation. C-shaped C. crescentus cells with pili showed discrete peaks of angle *α* at 180° and at 150° (Fig. 2B). In comparison, a Δ*creS* mutant that lacks the characteristic crescentoid cell curvature (47) showed only a single peak at 180°. This indicated that the peak at 150° represents cells with their natural, unstrained crescentin-mediated curvature in the late predivisional stage. A mutant lacking pili did not show a peak at 180° but retained peaks at 150°, arguing that cell stretching before division is mediated by the action of polar pili bound to a surface. Consistent with this, cells lacking the polar flagellum retained their stretching ability (Fig. 2B). Moreover, cell stretching was more prominent at very late stages of division, with the peak at 180° increasing at the expense of the peak at 150° (Fig. 2B). Finally, we observed a striking difference between the behavior of predivisional cells that generated offspring able to attach after separation (Fig. 2B, attach) and that of predivisional cells producing SW cells that were washed out after cell division (Fig. 2B, wash out). While the former showed a prominent peak at 180°, this peak was missing in dividing cells destined to produce offspring unable to attach. Instead, the latter showed a distribution of angle *α* resembling the pilus-deficient strain. The correlation of pilus-mediated predivisional cell stretching and successful surface attachment of SW offspring suggested that pili play an active role in surface attachment. This is consistent with the observation that SW offspring, which were freely rotating before detachment from their mothers, generally failed to remain attached to the surface (28). Of note, the orientation of the concave side of attached predivisional cells showed a strong bias to one side with respect to the flow direction (Fig. 2B; see also Fig. S2A to C). This suggested that the “C” shape of C. crescentus cells actually has a small helical twist (Fig. S2). While the concavity distribution is expected to be random for cells with a straight C shape, we found that the cells were more likely to orient to the left with respect to the flow direction, irrespective of their position within the channel or the nature of the surface.

10.1128/mBio.01237-19.2 FIG S2 (A) Bright-field images (left) showing C. crescentus cells attached to the floor and ceiling of a microfluidic channel. Images were analyzed with a MATLAB-based script to determine the cells’ concavity orientation (right). Cells with the concavity oriented to the right (red) or to the left (blue) with respect to the direction of the flow are highlighted. (B) Schematic drawing of the proposed cell shape of crescentoid C. crescentus with an exaggerated left-handed twist. (C) Quantitative analysis of concavity orientation (*n* = 3) with cells scored in an area of ≥0.3 mm^2^ on both the ceiling and floor surfaces of a device. The concavity distributions were identical in the right half (Right) and the left half (Left) of the channel, excluding flow-related effects. Download FIG S2, PDF file, 0.3 MB. Copyright © 2019 Sangermani et al. 2019 Sangermani et al. This content is distributed under the terms of the Creative Commons Attribution 4.0 International license.

Together, these experiments indicated that pili are assembled and active at the flagellate pole before cell division takes place and that pilus activity increases as dividing cells approach the separation stage. Moreover, dynamic pili are instrumental for surface attachment. We had proposed earlier that pilus retraction at this stage of cell division is critical to position the flagellar mechanosensor in close proximity to the surface in order to successfully initiate biogenesis of the adhesive holdfast, therefore preventing cells from being washed out (28).

### Dynamic Tad pili pull attached swarmer cells into an upright position.

We observed that within a few seconds after cell division, newborn SW cells were standing up against the medium flow. To quantify these movements, we measured the two-dimensional (2D) projections of individual cells in the *xz* plane and used this information to infer the cells’ 3D orientation and tilt angles (θ) over time (Fig. 2C). Newborn SW cells were unable to change their position as long as they were physically connected to their ST mothers. However, upon separation, SW cells rapidly changed their tilt, moving into an upright position of about 60 to 75° degrees. In contrast, ST cells retained their low θ value after cell division (Fig. 2D and E). Because mutants unable to assemble pilus fibers (Δ*pilA* mutants) showed very low levels of attachment, we analyzed the cell movements of a strain lacking both the major pilin subunit and the external parts of the flagellum (mutant Δ*flgDE*) as a control. We had shown earlier that C. crescentus cells lacking the outer parts of the polar flagellum (Δ*flgDE* cells) show a hypersensitive surface response that partially alleviates the strict requirement for pili (28). In agreement with this, SW cells of the Δ*flgDE* Δ*pilA* mutant sporadically remained surface attached after separation from their mothers. Importantly, all cells invariably retained the same low θ angle value (Fig. 2D). In contrast, a strain lacking only the flagellar structures (strain Δ*flgDE*) showed wild-type (*wt*)-like standing-up behavior upon cell separation (Fig. 2D and E). Deletion of the crescentin cytoskeleton (Δ*creS*) did not influence the ability of attached cells to stand up, arguing that cell curvature does not influence dynamic cell movements after division. Taking the data together, this indicated that force-generating pili are required for newborn SW cells to immediately move into a vertical (upright) position. Despite the relatively strong medium flow, daughter cells were able to keep their upright position for about 10 to 15 min before angle θ gradually decreased. This coincides well with the timing of the differentiation of swarmer cells to stalked cells that occurs when pili disappear and stalk biogenesis is initiated (Fig. 1B).

### Tad pili mediate walking movements against the medium flow.

Generally, pilus-mediated cell movements into an upright position or predivisional cell stretching were observed only once whereas repeated cycles of pilus extension and retraction were observed occasionally. Moreover, we occasionally observed cells moving for very short (1-to-3-μm) distances in holdfast-deficient cells after landing on a surface. We reasoned that the prevailing activity of pili might be masked in attached SW cells by the rapid synthesis of the holdfast adhesin, which immobilizes cells in an upright position (28).

**FIG 3:**
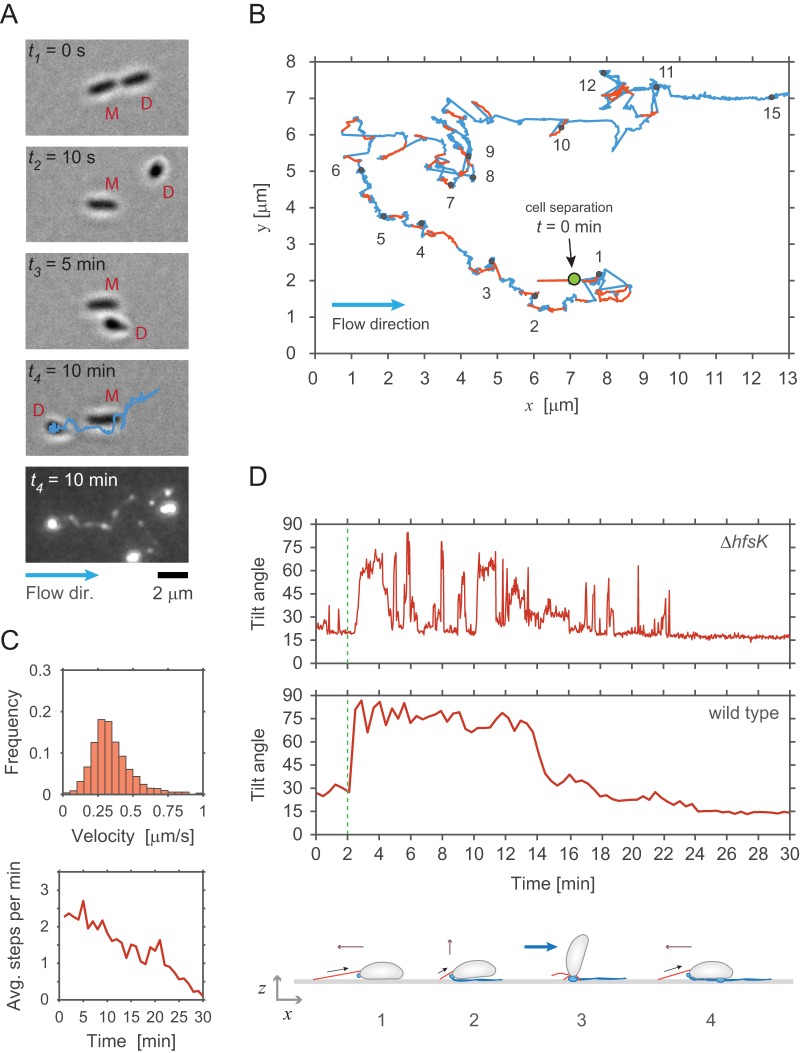
Dynamic pili assist walking-like movements against the medium flow. (A) Example of a newborn SW cell of a Δ*hfsK* mutant moving against the medium flow. The secretion of holdfast adhesin is monitored microscopically by employing fluorescently labeled wheat germ agglutinin (bottom image). The time after cell division is indicated, and mother (M) and daughter (D) cells are individually labeled. Time points *t*_1_ and *t*_2_ show the SW cell immediately before and after separation from its mother. Time points *t_3_* and *t_4_* show how the SW cell moves against the medium flow (blue arrow) past its mother. The blue track, which indicates the trajectory of the cell recorded during its 10 min walk, perfectly matches trails of holdfast material left behind. (B) Representative trajectory (blue line) of a SW cell moving on the surface of a microfluidic channel. The trajectory is reconstructed from time-lapse images recorded for a single cell of the Δ*hfsK* mutant. Step events were identified as fast movements against the flow and are highlighted in red. Black dots in the track indicate the time (minutes) after cell separation. (C) Pilus-mediated walking speeds (upper chart) and average number of step events per minute (lower chart) recorded for SW cells of the Δ*hfsK* mutant at the time points indicated after cell division (time zero) (*n *=* *56). (D) SW cells were repeatedly standing up during movements against medium flow. The tilt angle *θ* was recorded over time for representative examples of walking SW cells of a Δ*hfsK* mutant (upper panel) and the wild type (lower panel). A schematic of walking movements of the Δ*hfsK* mutant is shown below the charts. Retraction of an extended pilus pulls a horizontally positioned cell forward (step 1). Upon full retraction of the pilus, the cell body is pulled into an upright position (step 2), against the drag force of the medium flow (step 3). Upon completion of pilus retraction, the cell is pushed back onto the surface by flow (step 4) followed by the next motility step catalyzed by an extended pilus.

To address if C. crescentus pili are capable of undergoing multiple cycles of extension and retraction, we made use of a *hfsK* mutant (35). HfsK is a c-di-GMP effector protein, the activity of which increases holdfast cohesion. Mutants lacking this protein secrete holdfast material that is strong enough to glue cells to the surface in microfluidic devices with medium flow but that fails to firmly anchor cells at the place of initial attachment (35). To tune the amount of holdfast generated, a Δ*hfsK* strain was engineered that allowed modulating intracellular levels of c-di-GMP (*rcdG^0^*::*P_lac_-dgcZ*), the primary allosteric regulator of the holdfast secretion machinery, via the controlled expression of the exogenous diguanylate cyclase gene *dgcZ* (28). In flow chambers, cells of this strain were dragged across the surface by the medium flow, leaving trails of stained holdfast material behind (35). Intriguingly, we observed that newborn SW cells, after standing up, were able to move against the medium flow (Fig. 3A and B). These results indicated that pili can retain their dynamic behavior to dislodge cells that are weakly attached to the surface. Tracking the trajectories of individual cells identified the time period, step size, and speed of pilus-driven movements (Fig. 3B). On average, moving cells reached a speed of about 300 nm/s, covering distances of up to 500 nm in individual steps (Fig. 3C; see also Fig. S3A). Overall, cells covered distances of several micrometers in repetitive small steps with a frequency of about 2 to 3 steps per min (Fig. 3C). While the step frequency remained high for the first 10 to 15 min after division, it gradually decreased over time and the steps discontinued about 20 to 30 min after daughter cells had separated from their mothers (Fig. 3C). During their movements, SW cells, although unable to remain standing for longer periods of time, repeatedly moved back into an upright position (Fig. 3D). This behavior was particularly pronounced at the conclusion of each step event, arguing that full retraction of pili forces cells into an upright position (Fig. 3D; see also Fig. S3B). Together, these results indicated that C. crescentus pili remain highly active over longer periods of time and that, under these conditions, they engage in repetitive cycles of extension and retraction. This is highly reminiscent of the twitching or walking movements observed for other bacteria possessing type IV pili (18, 48).

10.1128/mBio.01237-19.3 FIG S3 (A) Step length distribution of C. crescentus Δ*hfsK* mutant cells during surface motility (*n *=* *56) (upper panel) and distribution of pilus lengths in wild-type cells as observed by TEM (*n *=* *271) (lower panel). (B) Schematic of a C. crescentus SW cell moving against the medium flow and standing upright at the end of each dislocation step. Pili (red), holdfast (blue), and cell movement (red arrow) are indicated. The charts below the graph show the distributions of tilt angle values 5 s before (left), during (middle), and 5 s after (right) an upstream step event. The cells were more likely to lie flat on the surface before and during a step event and to stand up upon completion of an upstream movement. Download FIG S3, PDF file, 0.8 MB. Copyright © 2019 Sangermani et al. 2019 Sangermani et al. This content is distributed under the terms of the Creative Commons Attribution 4.0 International license.

### Determining the force and energy of pilus retraction.

**FIG 4:**
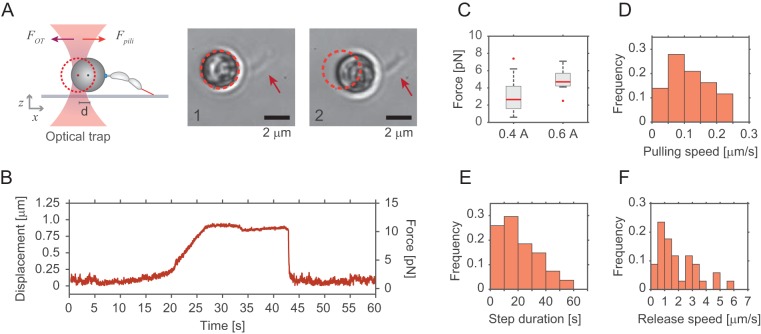
Optical tweezers determine pilus retraction force and speed. (A) Schematic representation of the experimental setup used for optical trap measurements of pilus retraction forces. Beads with late predivisional cells attached via their holdfast were trapped and maneuvered toward the surface to allow pili extending from the opposite pole (red) to attach. Upon pilus retraction, bead displacement was measured. Images on the right show a representative example of a bead with one attached predivisional cell (arrow) before (image 1) and after (image 2) retraction. Note that the trapped bead was displaced by about 1 μm. (B) Pilus-mediated displacement of trapped beads over time. The plot is representative of an optical tweezer measurement, showing the displacement and the respective forces generated by pilus retraction. (C and D) Force and pulling velocity measurements of pilus retraction. (C) Median values (red line) and quartiles (boxes) of force generated by pilus retraction. Outliers are plotted as red points. The measurements were conducted with the laser power of the optical trap maintained at either 0.4 A (*n *=* *100) or 0.6 A (*n *=* *11). (D) Speed of retraction of individual pili with the laser power set at 0.4 A. (E and F) Step duration and pilus release time. (E) Durations of pilus retraction were quantified as the time from the start of bead displacement to the moment of bead release (*n *=* *28). (F) Speed with which the bead moved back into the center of the trap at the end of individual step events (*n *=* *34).

To measure the forces generated by pilus retraction, we made use of optical tweezers. Because C. crescentus cells are highly susceptible to the phototoxicity elicited by the strong light source used in optical traps, it was not practical to directly manipulate cells with this method. To avoid this problem, we exploited the ability of C. crescentus predivisional cells to permanently adhere to surfaces via their adhesive holdfast. By coupling predivisional cells to polystyrene beads and immobilizing the beads in the optical trap, phototoxicity was eliminated, as cells could now be kept in the optical trap for at least 1 h without losing their ability to grow and divide (Fig. 4A). By moving the beads carrying predivisional cells toward the glass surface, pili were able to attach and, upon retraction, displace the beads from the trap (Fig. 4A).

To avoid potential interference from the flagellum, these measurements were carried out with a strain lacking the external parts of the rotary motor (strain Δ*flgDE*). A representative example of an optical trap measurement is shown in Fig. 4B. Initially, the bead rests in the center of the optical trap, with some noise due to Brownian motion. Upon pilus attachment to the surface, Brownian motion decreases followed by bead displacement of up to 1 μm from the center before a rapid movement back into its original position (Fig. 4B). Bead displacement (*d*) and trap stiffness (*Κ*_trap_) values allowed calculation of the maximum retraction force (*F* = *Κ*_trap_·*d*). We tested different *Κ*_trap_ values and found a maximum retraction force applied by pili during retraction of approximately 8 pN (Fig. 4C). These values are slightly lower than those reported earlier, in which pilus forces were assessed by measuring the displacement of elastic micropillars (27). These values are similar to the drag force of around 10 pN that surface-bound cells experience in a microchannel with a medium flow speed of 0.75 mm/s (Fig. S1B). Other bacteria such as P. aeruginosa or N. gonorrhoeae with type IVa pili display higher pilus retraction forces (30 to 100 pN) (4, 5, 49), possibly reflecting lower drag forces experienced by C. crescentus in natural settings and differential functionalities of type VIc pili related to rapid holdfast-mediated attachment.

The mean speed of C. crescentus Tad pilus retraction was 100 nm/s (Fig. 4D). In contrast, the speed at which beads backtracked into the trap center was considerably higher (Fig. S4E and F), arguing that these events resulted from pilus detachment rather than reelongation of the filaments. From this, we concluded that elongation events are unlikely to interrupt retraction phases, suggesting that pilus retraction is processive, showing disassembly without interruption until all pilin subunits are internalized.

**FIG 5:**
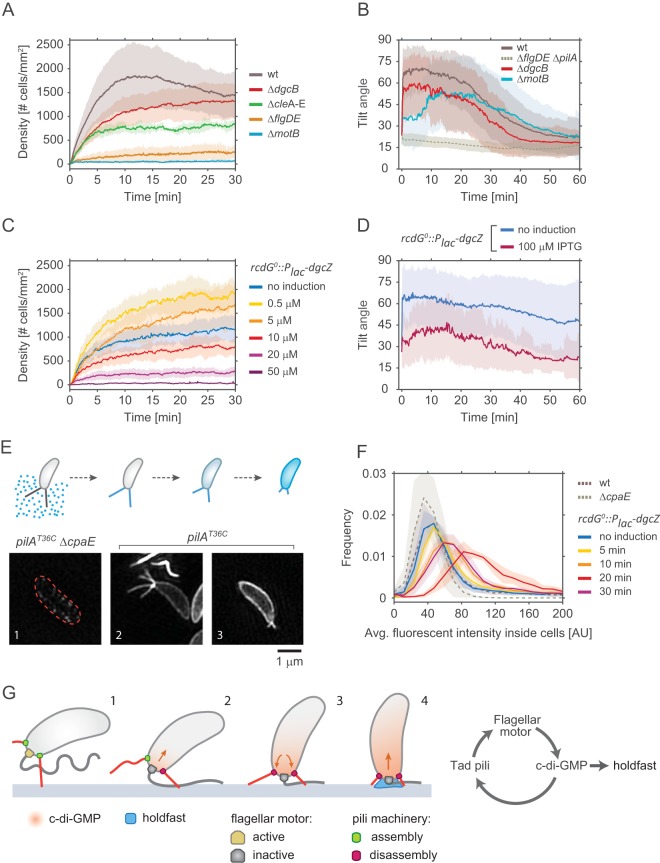
Effect of c-di-GMP on pilus activity and surface attachment. (A) Pilus-mediated surface attachment in different strains of C. crescentus strain. The colonization density was determined over time in a microchannel at a constant medium flow rate of 0.75 mm/s. All strains used were defective in holdfast secretion (NA1000). Shadow areas represent standard deviations. Number of replicates: wt strain = 14, strain Δ*dgcB* = 14, strain Δ*flgDE* = 6, strain Δ*motB* = 10, strain Δ*cleA–E *=* *6. (B) Pilus-mediated standing up of SW cells. The tilt angle *θ* was determined in newborn SW cells of the strains indicated. Time zero corresponds to the moment of SW cell separation from its mother. Shadow areas represent standard deviations. All strains had a functional holdfast. Number of replicates: wt strain = 96, strain Δ*motB* = 54, strain Δ*dgcB* = 34, strain Δ*pilA* = 15. (C) Pilus-mediated attachment efficiency as a function of the presence of or absence of c-di-GMP. Strain NA1000 *rcdG^0^*::*P_lac_-dgcZ* was grown at increasing IPTG concentrations to raise intracellular c-di-GMP and was analyzed for surface colonization as outlined for panel A. Shadow areas represent standard deviations. Number of replicates: no induction = 8, 0.5 μM = 6, 10 μM = 6, 20 μM = 4, 50 μM = 6. (D) Pilus-mediated standing up of SW cells as a function of c-di-GMP concentration. The angle *θ* was determined for newborn SW cells of strain *rcdG^0^*::*P_lac_-dgcZ* after cell division. Two distinct *dgcZ* expression levels were tested: no induction (*n *=* *44) and 100 μM IPTG (*n *=* *41). Shadow areas represent standard deviations. (E) The upper schematic shows the labeling process for pili. Fluorescent maleimide dye in the medium can label only exposed cysteines, such as those in elongated pili carrying mutated *pilA*^T36C^ pilin subunits. When labeled pili were retracted, pilin subunits diffused in the cell membrane, and the fluorescent signal correlates with the amount of pilin subunits in the membrane. The lower part of the panel shows representative superresolution images of labeled cells. (Step 1) Strains that carry *pilA*^T36C^ but that are unable to assemble pili due to a mutation in the motor machinery (Δ*cpaE*) do not acquire any significant fluorescence. (Step 2) In cells carrying *pilA*^T36C^ and a functional machinery, elongated pili are strongly labeled. (Step 3) Upon retraction, disassembled pilin subunits reside in the membrane. (F) The chart shows the distribution of the average fluorescent signal of SW cell membranes in different strains carrying the mutation *pilA*^T36C^ after labeling with AF488-mal for exact incubation time windows. (Replicates = >3; analyzed cells = >6,000). Note that because AF488-mal nonspecifically binds to holdfast material, all strains used here were devoid of holdfast. (G) Model of surface attachment and transition from temporary to long-term attachment. Within a few seconds of landing, cells sense the surface via the flagellar motor and increase their levels of intracellular c-di-GMP. In turn, c-di-GMP triggers the secretion of the holdfast and increases the rate of retraction of pili.

10.1128/mBio.01237-19.4 FIG S4 (A) Surface attachment of SW cells of different wild-type and mutant strains in microfluidic devices. Average numbers of newly attached cells per square millimeter per second are shown in the upper panel. The lower panel shows desorption frequencies of the same strains, calculated as the ratio of the number of cells leaving the surface to the total number of cells attached between two time points (5 s). Values were obtained from the attachment assays shown in Fig. 5A and C during the time window between min 10 and min 25. Error bars indicate standard deviations. (B) Residence time of cells on surfaces during pilus-mediated attachment. Each curve indicates the cumulative fraction of cells residing on a surface for a period equal to or greater than the indicated time. Opaque areas represent standard deviations. All strains were unable to secrete holdfast (NA1000). Number of replicates: upper chart, >5; lower chart, >4. (C) Scatter plots with the average angle θ representing SW cells (red) and ST cells (gray) recorded 5 min before and 5 min after cell separation. Number of replicates: strain Δ*motB* = 41; strain Δ*dgcB* = 45; strain *rcdG^0^*::*P_lac_-dgcZ* (0 M) = 50; strain *rcdG^0^*::*P_lac_-dgcZ* (1 μM) = 46. (D) Number of pili observed at the pole of individual C. crescentus wild-type cells imaged by TEM. In the experiments represented in the upper chart, wild-type cells were fixed either before (planktonic) or after being spotted on EM grids for 5, 10, and 20 min (surface) to allow them to make surface contact. The fractions of cells with specific numbers of pili are indicated. The lower chart shows pilus numbers in strain *rcdG^0^*::*P_lac_-dgcZ* at different levels of IPTG induction. In this case, cells were fixed 5 min after making surface contact. (E) Representative images of different C. crescentus strains after pilus labeling. Strains engineered to express the *pilA^T36C^* allele were specifically labeled with the fluorescent dye AF-647-mal. Strains expressing a wild-type *pilA* allele or defective in pilus assembly (*ΔcpaE*) were used as controls. Images were acquired by fluorescence microscopy. Bright-field (gray-scale) and fluorescence images are overlaid (red). The fluorescent channels of the different images were set to the same parameters. All strains used were unable to secrete holdfast (NA1000). (F) Immunoblot analysis of C. crescentus wild-type and mutant strains using an antibody against the major pilin subunit PilA. Strain *rcdG^0^*::*P_lac_-dgcZ* was tested without IPTG induction or in the presence of 100 μM IPTG for different time windows. C. crescentus wild-type (wt) and Δ*pilA* mutant samples were used as controls. Download FIG S4, PDF file, 0.7 MB. Copyright © 2019 Sangermani et al. 2019 Sangermani et al. This content is distributed under the terms of the Creative Commons Attribution 4.0 International license.

### C-di-GMP regulates Tad pilus activity.

To better understand the role of pili during surface attachment and their functional interaction with the flagellum, we scored the colonization efficiency of nonmotile flagellar mutants in microfluidic channels, using the same setup as described above (Fig. 2). Both the Δ*motB* and the Δ*flgDE* mutants showed very low levels of colonization densities, suggesting that active swimming is important for cells to efficiently reach the surface in this experimental setup (Fig. 5A). Interestingly, nonmotile strains also showed higher detachment frequencies and shorter residence times than the wild-type strain (Fig. S4A and B), behaviors that were most pronounced for the Δ*motB* strain. Since the MotA and MotB stator units of the flagellar motor are involved in surface sensing (28), this indicated that the flagellar motor and pili may be connected through a regulatory-feedback mechanism.

It was recently shown that C. crescentus surface sensing leads to a rapid increase in the level of the second messenger c-di-GMP, which in turn mediates biogenesis of the adhesive holdfast (27, 28). One of the enzymes implicated in this process is the diguanylate cyclase DgcB (28). However, it has remained unclear if Tad pili are positioned upstream of c-di-GMP and if they contribute to the increase in the level of the second messenger during mechanotransduction or if Tad pili are a target of c-di-GMP control during this process. To clarify this, we monitored the behavior of pili in response to changing levels of c-di-GMP. Intriguingly, a Δ*dgcB* mutant showed a significant delay in pilus-mediated surface colonization (Fig. 5A) and a minor defect in standing up against the medium flow (Fig. 5B) compared to wild type. The fact that pilus-mediated behavior was affected only partially in the Δ*dgcB* mutant in these experiments indicated that an additional diguanylate cyclase(s) may be involved in this process (28). This is in line with the observation that newborn SW cells of a *ΔmotB* mutant that were unable to sense surface showed a considerably stronger defect in reorienting into an upright position against the medium flow (Fig. 5B), while a Δ*flgDE* mutant (without a surface-sensing defect) was not affected (Fig. 2E). In agreement with the view that Tad pili are able to respond to changes in c-di-GMP, we found that the level of pilus-mediated attachment was strongly reduced in a mutant lacking several CheY-like Cle proteins (Fig. 5A). Cle proteins bind c-di-GMP and then can interact with the flagellum and are an integral part of C. crescentus surface recognition (50).

To analyze the role of global c-di-GMP levels in pilus dynamics, we tested a strain harboring an inducible copy of the *dgcZ* diguanylate cyclase gene (*rcdG^0^*::*P_lac_-dgcZ*). While low-level induction of *dgcZ* increased pilus-mediated surface attachment, surface colonization gradually decreased upon stronger induction of *dgcZ* (Fig. 5C). This effect was the consequence of decreased attachment frequencies (Fig. S4A). Low-level induction of *dgcZ* also decreased detachment rates and increased the cells’ residence time on surface; however, these effects were reversed at higher levels of induction of *dgcZ*. Furthermore, increased induction of *dgcZ* also strongly interfered with the ability of newborn SW cells to stand up (Fig. 5D; see also Fig. S4C). These observations are in line with an earlier report demonstrating that a low level of c-di-GMP is strictly required for C. crescentus Tad pilus expression and assembly (32). Transmission electron microscopy (TEM) revealed that the number of pili per SW cell increased upon surface exposure and that piliation was indeed dependent on the intracellular c-di-GMP concentration. While low-level induction of *dgcZ* increased piliation, the number of pili per cell decreased at higher *dgcZ* expression levels (Fig. S4D). Together, these results indicated that both the flagellar motor and c-di-GMP control Tad pilus-mediated behavior, arguing that c-di-GMP is positioned upstream of pili in the mechanotransduction pathway and is able to regulate pilus dynamics.

### High concentrations of c-di-GMP promote Tad pilus retraction.

The results described above suggested that high levels of c-di-GMP promote Tad pilus retraction. To directly visualize Tad pilus dynamics, we used a *pilA*^T36C^ gene to fluorescently label pilus subunits with maleimide-based fluorescent dyes. This technique was recently used to visualize Tad pilus retraction in real time (27). Although we were able to visualize sporadic events of pilus retraction, pilus filaments are fragile and are prone to breaking during the labeling process. Because only a very few cells showed dynamic pili after labeling, it was difficult to obtain statistically solid data sets to compare the behaviors of different mutant strains. To overcome this problem, we adopted a more static fluorescence assay, with *pilA*^T36C^ pilus labeled externally by fluorescence maleimide dyes. Because the fluorescent dyes are unable to penetrate the cell envelope, PilA subunits are labeled only if they are assembled into a pilus filament, thereby crossing the outer membrane (27) (Fig. 5E). Upon pilus retraction, disassembled, now fluorescent PilA subunits diffuse back into the cytoplasmic membrane, thereby increasing the fluorescence signal in this compartment (Fig. 5E) (27). Thus, to enable quantification of pilus retraction, we determined the fluorescence intensities of cell membranes rather than directly monitoring the fragile exterior pilus filaments. Importantly, cells expressing wild-type *pilA* or cells expressing *pilA*^T36C^ but lacking the motor subunit of the pilus machinery (Δ*cpaE*) did not accumulate fluorescence. In contrast, cells expressing *pilA*^T36C^ showed a weak but robust fluorescence signal (Fig. 5E and F; see also Fig. S4E). Similarly, a strain harboring *dgcZ* (strain *rcdG^0^*::*P_lac_-dgcZ*) showed weak fluorescence when expression of the exogenous diguanylate cyclase was not induced (Fig. 5F; see also Fig. S4E). To monitor Tad pilus dynamics at increasing c-di-GMP concentrations, *dgcZ* expression was induced for increasing amounts of time before external pili were labeled by adding the dye. When *dgcZ* expression was induced for 5 min, the average fluorescence intensity of cells slightly increased compared to uninduced cells. Longer induction of *dgcZ* for 10 or 20 min gradually increased the amount of fluorescence accumulating in the cytoplasmic membrane in a high proportion of cells (Fig. 5F; see also Fig. S4E). Thus, as c-di-GMP levels built up, Tad pilus activity increased. However, when *dgcZ* was induced for more than 20 min before pili were labeled externally, fluorescence in the cytoplasmic membrane decreased, indicating reduced pilus dynamics under these conditions. Importantly, *dgcZ* induction did not affect the overall concentration of pilin subunits (Fig. S4F). These results support a model where moderate c-di-GMP levels increase pilus dynamics whereas higher c-di-GMP levels lead to the retraction of Tad pili (Fig. 5G).

## DISCUSSION

Bacteria have evolved complex mechanisms that enable them to effectively colonize surfaces through a combination of tactile sensing and the exposure of surface adhesins. We showed earlier that C. crescentus SW cells are able to sense surface encounter with their polar flagellar motor and, in response, to deploy an adhesive holdfast to remain irreversibly anchored on the surface. On the basis of the results presented here, we propose that the spatially associated flagellum and Tad pili synergistically optimize the C. crescentus surface response. The succession of events leading from temporary to long-term attachment is summarized in the model in Fig. 5G. SW cells swimming in close proximity to a surface are able to attach via preexisting Tad pili (Fig. 5G, step 1), thereby creating opportunities for initial surface sensing by the flagellar motor (Fig. 5G, step 2). Activation of the flagellar-motor-coupled diguanylate cyclase DgcB (and possibly other diguanylate cyclases) then generates an increase in the level of c-di-GMP, which boosts the activity of Tad pili. The assembly of additional polar pili increases the cell’s probability of remaining attached to and of forming tight connections with the surface (Fig. 5G, step 3). As c-di-GMP levels increase, processive pilus retraction nudges the flagellate pole into close proximity to and contact with the surface. In particular, reorientation of cells into an upright position optimally positions the flagellar motor, strengthening its tactile sensing and further increasing c-di-GMP to peak levels required for the allosteric activation of holdfast biogenesis (Fig. 5G, step 4) (28). Our model proposes that the role of pili goes beyond that of passive adhesins promoting temporary attachment and that, instead, Tad pili and the flagellar motor together promote surface adherence in a highly dynamic and coordinated fashion. The model predicts that Tad pili actively guide the efficient surface sensing by the flagellar motor and thus contribute to the critical upshift of c-di-GMP levels mediated by DgcB and by other, as-yet-unknown diguanylate cyclases. This model is compatible with the notion that Tad pili themselves may contribute to surface sensing during this process (27).

Evidence for our model comes from direct observation of Tad pili, which increase in numbers when cells are surface exposed or when they experience a moderate increase of c-di-GMP levels but a decrease in numbers when c-di-GMP reaches peak levels. Moreover, incorporation of fluorescently labeled pilin subunits indicated high Tad pilus activity at intermediate levels of c-di-GMP but reduced pilus activity when levels of c-di-GMP further increased. Finally, pilus-mediated surface attachment and vertical positioning of cells were abrogated when c-di-GMP levels were artificially increased to higher levels. On the basis of these observations, we speculate that at moderate levels of c-di-GMP, the overall number of polar pili per cell increases through a boost in assembly or through slowed disassembly or a combination thereof. In contrast, peak levels of c-di-GMP, which are reached upon sustained surface sensing or during development of the motile SW cell into a sessile ST cell (32, 51), signal pilus retraction and PilA subunits are internalized. Thus, the functional interdependence of the motor, c-di-GMP, and pilus retraction generates a positive-feedback loop that imposes a directional process that progressively drives cells toward holdfast-mediated permanent surface attachment (Fig. 5G). We had shown earlier that pili are not required *per se* for the motor-mediated surface response and that bacteria grown in very narrow microfluidic chambers where they constantly encounter surface are able to rapidly attach via their holdfast even if they lack pili (28). Under normal conditions, however, bacteria swimming close to a substratum experience surface contact only transiently, a situation that may fashion the need for a highly dynamic and efficient process to stabilize and reinforce this interaction on short time scales. Pili colocalize with the flagellum at one cell pole and are thus optimally positioned to direct the motor toward the surface and to incite collisions that optimize the strength and duration of mechanosensing. Tad pili are present (20) and active (Fig. 2) in the predivisional cell immediately before cell division takes place at a time when the flagellum becomes fully operational (28). In line with the idea that pili are available to reinforce the motor-mediated surface program, their activity in the predivisional cell strictly correlates with the ability of SW offspring to permanently attach next to their mothers in strong flow (Fig. 2). Likewise, SW cells that freely rotate around their long axis during the process of separating from their stalked mothers, presumably because they fail to be dragged toward the surface by Tad pili, rarely manage to remain attached to the surface. In contrast, cells that stopped their rotation generally remained attached after budding off their mothers (28). These observations directly link pilus activity with flagellar obstruction and surface sensing.

The notion that c-di-GMP, depending on its concentration, influences pilus activity in distinct ways is supported by observations indicating that c-di-GMP promotes type IV pilus assembly in several bacterial species, including C. crescentus (32), V. cholerae (40, 41), and P. aeruginosa (36, 39). Moreover, increased and decreased levels of c-di-GMP were shown to impact type IV pilus-based motility in Myxococcus xanthus, arguing that the second messenger is required for but interferes with pilus function at increased concentrations (17). Similarly, polar Tad pili are retracted during the C. crescentus SW-to-ST transition coincident with c-di-GMP reaching peak levels in ST cells (5, 32, 51). Mechanistically, this complex regulation could result from the antagonistic activities of two effector proteins that bind c-di-GMP with different affinities to promote the assembly and disassembly of pilus filaments, respectively. Similar mechanisms were described in P. aeruginosa and X. campestris, where two c-di-GMP binding proteins, FimX and FimW, localize to the cell poles to modulate pilus formation. While the mode of action of FimW is still unknown, FimX facilitates pilus elongation during twitching at the leading cell pole by interacting with the assembly motor ATPase PilB (38, 39). Direct interaction of c-di-GMP with the pilus machinery was also shown in V. cholerae, where c-di-GMP binding by the motor assembly protein MshE promotes polymerization of pili in a dose-dependent manner (40, 41). Exactly how c-di-GMP influences both pilus assembly and retraction in C. crescentus remains to be shown.

Our model for C. crescentus surface attachment proposes tight functional cooperation of the flagellar motor and Tad pili. Evidence for this stems from the observation that pilus activity was reduced in mutants lacking the MotB stator unit or lacking all five Cle proteins (CleA to CleE) (Fig. 5A and B). C. crescentus Cle (CheY-like) proteins were recently shown to bind c-di-GMP and, in response, to interact with the flagellar motor to impede flagellar activity and promote surface adaption (50). It was proposed that one or several of these proteins are part of a positive-feedback loop that reinforces the motor response during surface sensing, thereby facilitating a rapid upshift of c-di-GMP levels and production of holdfast material, which takes over the requirement for pilus-mediated attachment. The reduced pilus-mediated attachment observed for the strain lacking *cleA* to *cleE* (Δ*cleA–E*) could thus be due to an insufficient raise in c-di-GMP or, alternatively, may implicate one of the Cle proteins in the regulation of pili directly, providing a mechanistic basis for the coordination of both organelles. Further studies are needed to clarify the role of Cle components in this process.

Experiments in flow devices demonstrated that C. crescentus Tad pili are highly dynamic and are able to promote twitching- or walking-like movements of C. crescentus SW cells (Fig. 3). For these experiments, we used a mutant lacking the HfsK N-acetyltransferase, an enzyme that was proposed to chemically modify holdfast material. A Δ*hfsK* mutant forms malleable holdfast structures that can facilitate the clinging of cells to surfaces but lack the adhesive strength required to hold cells in place under flow conditions or when pulled by other forces such as pilus retraction. It was recently proposed that HfsK acylates the EPS component of the holdfast and that this modification is necessary for proper holdfast cohesion and anchoring (35). The observation that HfsK activity is itself regulated by c-di-GMP indicated that holdfast material may be formed under certain conditions but remains in a nonacylated form. If so, limited cohesive strength of the holdfast could attach C. crescentus cells to surfaces without restricting their pilus-mediated motility. This would allow this aquatic organism to explore liquid exposed surfaces with their pilus motors in a manner similar to the well-known twitching and walking motility of soil bacteria.

## MATERIALS AND METHODS

### Bacterial strains and growth conditions.

The wild-type (*wt*) strain of reference was either NA1000, a laboratory-adapted strain with a point mutation in gene *hfsA*, which makes it unable to secrete the holdfast, or NA1000 *hfsA^+^*, where the functional gene was reintroduced. The strain *rcdG^0^* is a c-di-GMP-free strain where all major endogenous GGDEF and EAL domain-encoding genes were deleted. Introduction in *rcdG^0^* of the exogenous diguanylate cyclase *dgcZ* gene under the control of the IPTG (isopropyl-β-d-thiogalactopyranoside)-inducible *lac* promoter (*P_lac_-dgcZ*) allows tuning of the expression of *dgcZ* and, in turn, control of the intracellular levels of c-di-GMP, as previously reported (32). A full list of the strains used in this study is provided in Table S1 in the supplemental material.

10.1128/mBio.01237-19.5 TABLE S1 Bacterial strains and plasmids used in this study. Download Table S1, DOCX file, 0.1 MB. Copyright © 2019 Sangermani et al. 2019 Sangermani et al. This content is distributed under the terms of the Creative Commons Attribution 4.0 International license.

### Fabrication of microfluidic devices.

Masters were fabricated via standard photolithography protocols (52). PDMS (polymethyldisiloxane [Sylgards 184]; Dow Corning) devices were created via replica molding and were then aged via heat treatment on a hotplate at 150°C for 30 min (53). Holes were drilled at the inlet(s) and the outlet(s), and the devices were treated in oxygen plasma and covalently bound onto round, 50-mm diameter borosilicate glass cover slides (VWR) (thickness no. 1). All microflow experiments were conducted in single microchannels with dimensions of either 100 μm in width and 25 μm in height or of 200 μm in width and 50 μm in height.

### Microfluidics and microscopy setup.

For attachment assays, overnight cultures (generally NA1000) were diluted 1:50 and grown at 30°C in peptone-yeast extract (PYE) medium with agitation. For the *rcdG^0^*::*P_lac_-dgcZ* strain, we added the desired IPTG concentration during the dilution step. Once the culture reached an optical density at 600 nm (OD_660_) of 0.15, it was loaded in a 1-ml plastic syringe (Soft-ject; Henke-Sass, Wolf). The syringe was then plugged into a needle (BraunMelsungen AG) (23 gauge, 0.6 by 30 mm) which was connected in turn to one end of a polytetrafluoroethylene (PTFE) microtube (Fisher Scientific) (0.56 by 1.07 mm). The tubing was filled with the culture, and the terminal end of the tubing was connected to the inlet of a microfluidic device (channel cross section, 50-μm width by 200-μm height). The syringe was mounted on a syringe pump (neMESYS low-pressure module V2; Cetoni GMBH) (14:1 gear). With the microfluidic setup ready, the device was placed on the stage of an inverted microscope (IX81; Olympus GMBH). The syringe pump was initially set to create a strong flow of 25 mm/s for 1 min to ensure that the microchannel surface was devoid of cells. The flow was then set to the desired target velocity at room temperature for the duration of the experiment (30 to 45 min). Time-lapse images were recorded at 0.1 to 0.16 frames/s (fps), using a 40× oil immersion objective (UPlanFLN 40× oil; Olympus). We used a new batch of culture at an OD_660_ of 0.15 for each experiment.

For observations of single-cell division events, a culture of the desired strain (generally *hfsA^+^*) was loaded in the microfluidic channel of a device (channel cross section, 25-μm width by 100-μm height). Cells were left to colonize the surface for a short period, resulting in an average distance between cells of about 20 to 30 μm. This low density ensured that single-cell division events were not disturbed or influenced by neighboring cells. A 1-ml plastic syringe loaded with fresh PYE medium (plus the desired IPTG concentration for strain *rcdG^0^*::*P_lac_-dgcZ*) was plugged into a needle and connected to one end of a PTFE microtube. The syringe was then mounted on a syringe pump and the terminal end of the tubing plugged into the device’s inlet. A steady flow of 1 mm/s was then set for the duration of the entire experiment. Cells were left to grow for 1 to 2 h before recording started with image sequences taken at 1 or 5 fps, using a 100× oil immersion objective (CFI Plan Apo λDM100× oil; Nikon). The experiments were conducted at room temperature for no more than 10 h. This procedure ensured steady growth conditions and no overgrowth/clogging in the inlet.

### Optical tweezer setup and force measurements.

The optical tweezer experiments were performed on a custom-built bright-field microscope, complemented with a laser diode setup (LD830-MA1W; Thorlabs) (λ = 830 nm). A water immersion, high-aperture objective (UPlanSApo 60× water; Olympus) was used to focus the laser beam, trap the beads with attached bacteria, and image the fluctuations of the bead in the trap and the attached bacteria. The experiments were carried out in position clamp mode at a constant laser power setting. The images of the beads and the attached cells were recorded at 50 Hz and 75 Hz using a fast camera (Phantom Miro EX4; Vision Research Inc.). We measured the change in position of the bead during the experiment, and we observed the active cell.

Calibration of the optical tweezer was carried out via fluctuation calibration. For each laser power setting used, an image sequence of a bead in the trap was recorded at 1,000 Hz. The variance (σ) was determined from the fluctuation of the bead in the trap. The trap stiffness (*Κ*_trap_) was calculated as follows: *Κ*_trap_ = *k_B_T*/*σ^2^*, with *k_B_* representing the Boltzmann constant and *T* the room temperature.

An exponential culture of strain NA1000 *hfsA*^+^ Δ*flgDE* maintained at an OD_660_ of 0.15 was mixed with polystyrene beads (Fluoresbrite YG Carboxylate Microspheres; Polysciences) (3.0-μm diameter) to reach a final concentration of 1.7·× 10^8^ beads per milliliter of cell suspension. The mix was incubated for 2 min to allow cells to attach to the beads. Then, a 1:1 dilution with fresh PYE medium was injected into the device and experiments were conducted under no-flow conditions. The devices used for optical tweezer measurements had chambers attached to a main channel (52, 54) connected by an opening of less than 10 μm. Single bead-carrying predivisional cells were chosen and placed inside the chambers where the optical tweezer measurements could be performed undisturbed from other cells.

### Pilus staining and fluorescence.

The labeling of pili was done following a protocol published recently by Ellison et al. (27). Briefly, an overnight culture was diluted 1:50 or 1:100 and grown to an OD_660_ of 0.10, with agitation. A 1-ml volume of the culture was transferred into an Eppendorf vial, 25 μg/ml of maleimide-reactive dye AF647-mal (Sigma-Aldrich) was added, and the reaction mixture was gently mixed by inversion. The sample was incubated for exactly 5 min and was then centrifuged (4,000 × g for 1 min), washed with 1 ml fresh PYE medium, and centrifuged again. The final pellet was resuspended in 50 μl fresh PYE medium. Finally, a 2-μl volume was spotted onto a 1% agarose pad and imaging at the microscope was conducted immediately. For strain *rcdG^0^*::*P_lac_-dgcZ*, we added 100 μM IPTG for an exact time window before the washing step. The total duration of IPTG induction included the 5-min maleimide-labeling step. For induction times longer than 5 min, the culture was kept in incubation until the labeling step.

The elapsed time between the washing step and the beginning of imaging was minimized, on average taking 4 to 5 min. Each sample was imaged for no longer than 7 to 8 min after the labeling step. Imaging was done at an inverted microscope (Eclipse Ti2; Nikon Instruments Europe B.V.) using a 100× oil immersion objective (CFI Plan Apo λDM100× oil; Nikon). We imaged 100 to 150 random positions for each pad, and we acquired a phase-contrast image (exposure, 50 ms) and a fluorescent image (excitation [Ex], laser wavelength of 640 nm at 25% intensity for 150 ms; emission [Em], mCherry 592 nm to 667 nm) for each position.

To analyze the fluorescent signal inside cells’ bodies, we used the MATLAB-based program microbeTracker for cell detection (55) and an in-house-built MATLAB-based program to analyze the fluorescent channel. We selected SW cells by using as the criterion a body length <2.5 μm.

### 3D-SIM superresolution microscopy.

Three-dimensional structured illumination microscopy (3D-SIM) was performed on a DeltaVision OMX-Blaze V4 system (GE Healthcare). Images were acquired using a Plan Apo N 60×, 1.42-numerical-aperture (NA) oil immersion lens objective (Olympus) and 4 liquid-cooled scientific complementary metal-oxide–semiconductor (sCMOS) cameras (pco.edge 5.5; PCO) (full frame, 2,560 by 2,160 pixels). Exiting light was directed through a movable optical grating to generate a fine-striped interference pattern on the sample plane. The pattern was shifted laterally through five phases and three angular rotations of 60° for each z-section. The 488-nm-wavelength laser lines were used during acquisition, and the optical z-sections were separated by 0.125 μm. Laser power was attenuated to 10%, and exposure times were typically 80 ms. The laser power was adjusted to achieve optimal intensities at between 2,000 and 3,000 counts in a raw image of 15-bit dynamic range at the lowest laser power possible to minimize photobleaching. Raw 3D-SIM images were processed and reconstructed using the DeltaVision OMX SoftWoRx software package (v6.1.3; GE Healthcare).

### Image analysis.

To quantify the attachment efficiency of C. crescentus SW cells in microfluidic devices, we used the MATLAB-based program microbeTracker for identification of surface-attached cells (55). The number of cells was assessed for every recorded frame of each experiment and normalized to the imaged area to obtain the cell density over time. To evaluate cell movements and orientations, we determined the cell outline, as detected by the MATLAB-based program microbeTracker (55). Further tracking, analysis, and statistics determinations were carried out with in-house-developed MATLAB scripts. Cell perimeters were fitted to an ellipse, and the eccentricities of the ellipses (ε), representing the ratio between the major and minor axes, were monitored. We used ε to calculate the inclination of cells with respect to the surface (angle θ). A ε value of 1 was set to correspond to a tilt angle (θ) of 90° and a ε value of 0.1 to correspond to a tilt of 0°. Eccentricity values below 0.1 could be excluded as they represent highly elongated ellipses, with a length-to-width ratio that does not occur for C. crescentus SW cells.

To track the trajectories of cells, we choose to follow the position of the holdfast. This position lies at a point between a vertex and a focus of the ellipse which fits the 2D projection of the cell. The position was dependent on the value of ε, such that when the cell is upright, it coincides with the focus, and when the cell lies flat, it is close to the vertex (see Fig. 2C). Step events (see Fig. 3B) were defined as representing fast movement against the flow, with speeds of ≥0.1 μm/s and movement sustained for ≥1 s.

### Electron microscopy.

To examine surface induced cells, 5-μl volumes of sample were spotted on a 400-mesh copper grid covered with a Parlodion and carbon film for 5 to 20 min. The grid was then gently washed using a micropipette, and cells were fixed with 0.1% glutaraldehyde. Alternatively, to examine planktonic samples, glutaraldehyde was added to a bacterial liquid culture to reach a final concentration of 0.1%. Then, 5-μl volumes were spotted on the grid. The samples were washed with water 4 times and subjected twice to negative staining with 0.5% uranyl acetate. Images taken at the TEM (Morgagni 268D FEI [80 kV] and FEI T12 [120 kV] TVIPS F416) were visually inspected and SW cells identified by the absence of a stalk and a cell length of <2.5 μm.

### Immunoblot analysis.

Proteins were separated by electrophoresis on 20% SDS-polyacrylamide gels and transferred onto a 0.2-μm-pore-size nitrocellulose blotting membrane (Amersham Protran 0.2-μm NC; GE Healthcare). A PageRuler prestained protein ladder (Thermo Fisher) was used to mark protein sizes. The primary antibody for detection was polyclonal rabbit anti-PilA antibody (56) (diluted 1:4,000). The secondary antibody was swine anti-rabbit antibody coupled to horseradish peroxidase (Dako), used at a dilution of 1:10,000. Antibody-treated blots were incubated with LumiGLO (KPL) for exposure of super RX-N films (Fujifilm).
