# Supplementary figures and images for: Tad Pili Play a Dynamic Role in Caulobacter crescentus Surface Colonization
Source: mBio. 2019 Jun 18;10(3):e01237-19. doi: 10.1128/mBio.01237-19 (PMC6581867; doi:10.1128/mBio.01237-19)

A

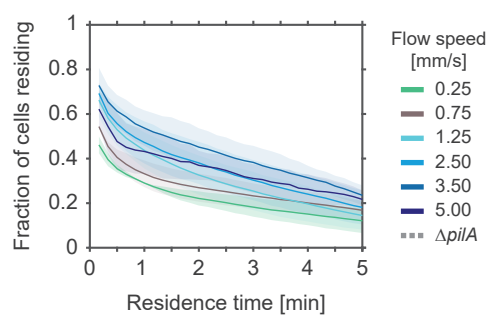

B

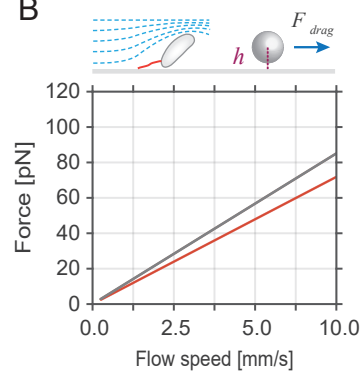

Supplement: FIG S1 [file mBio.01237-19-sf001.pdf]

A

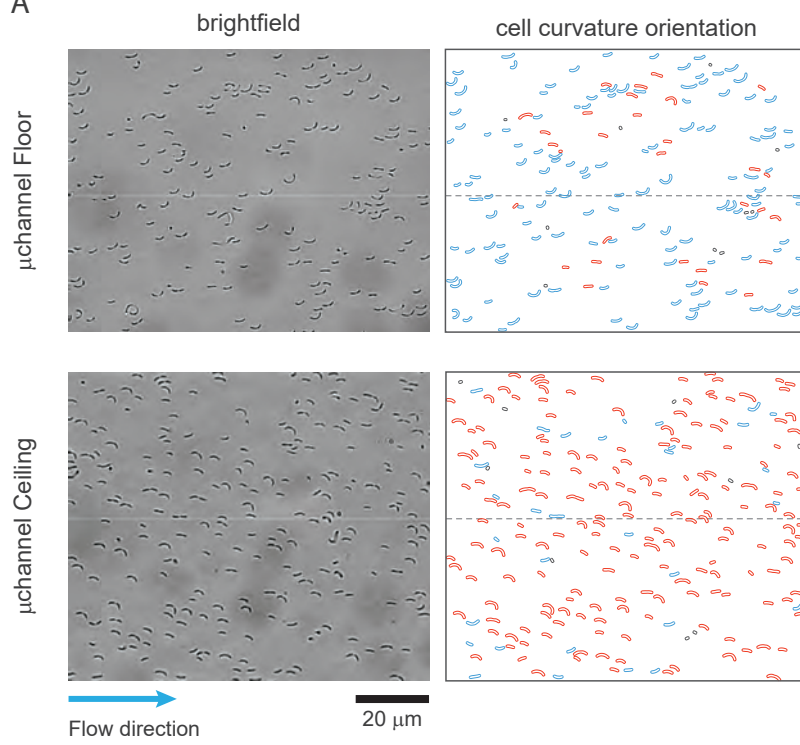

B

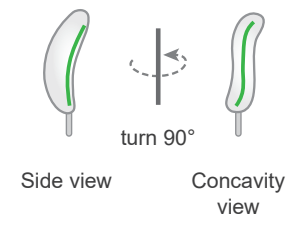

C

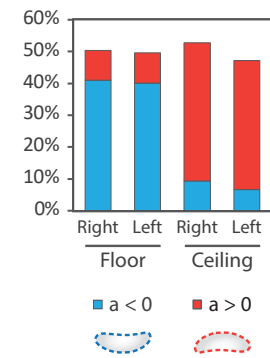

Supplement: FIG S2 [file mBio.01237-19-sf002.pdf]

A

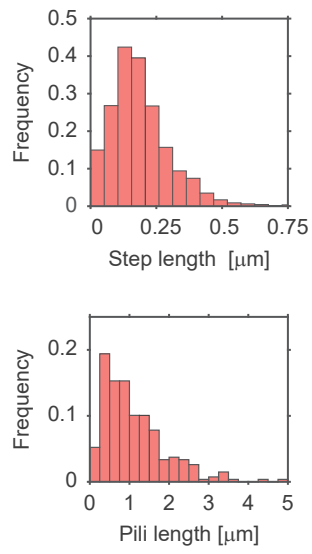

B

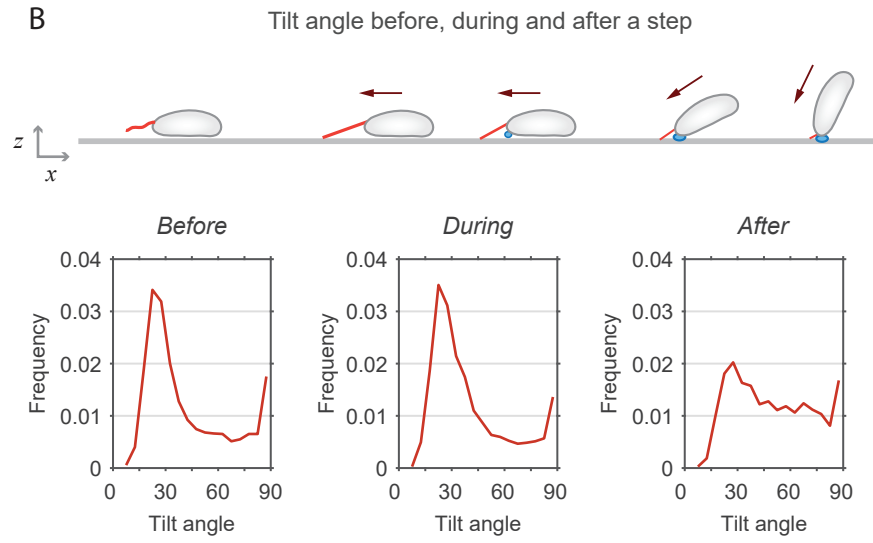

Supplement: FIG S3 [file mBio.01237-19-sf003.pdf]

A

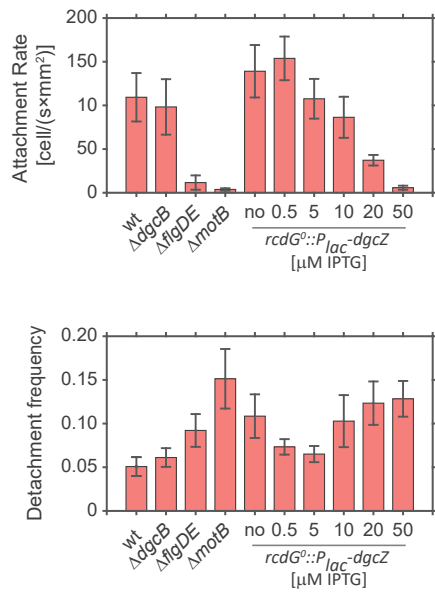

B

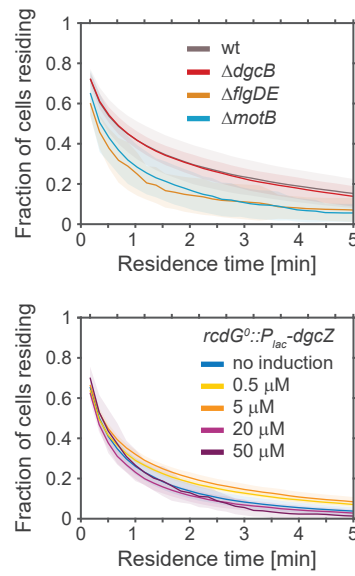

D

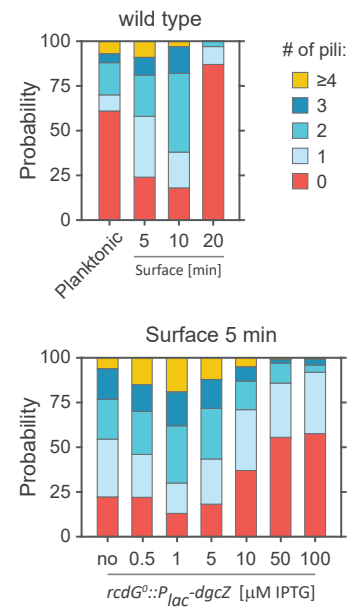

C

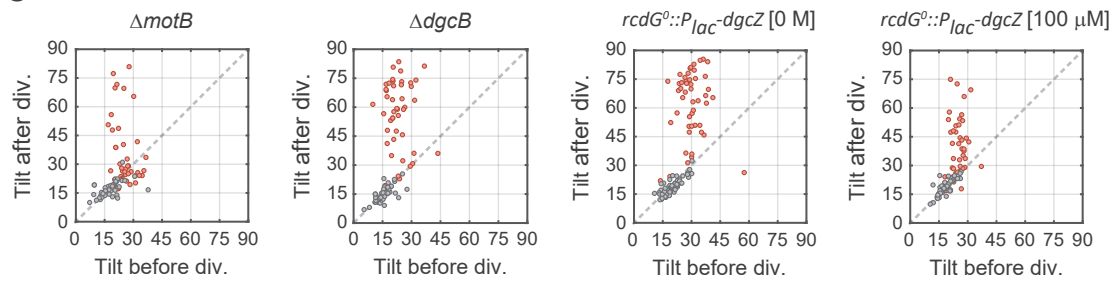

E

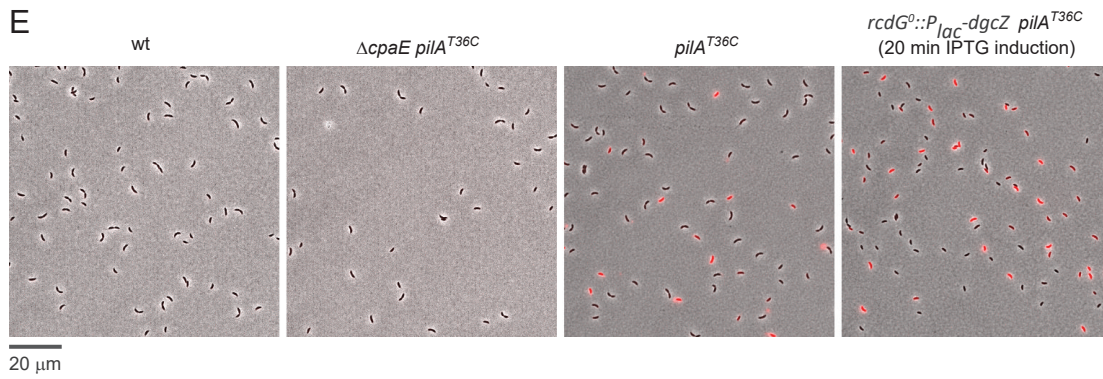

F

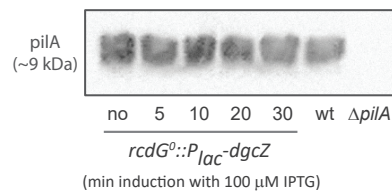

Supplement: FIG S4 [file mBio.01237-19-sf004.pdf]
