# Supplementary material for: Tad Pili Play a Dynamic Role in Caulobacter crescentus Surface Colonization
Source: mBio. 2019 Jun 18;10(3):e01237-19. doi: 10.1128/mBio.01237-19 (PMC6581867; doi:10.1128/mBio.01237-19)
Supplement: TABLE S1 [file mBio.01237-19-st001.docx]

Supplemental Table S1 - Bacterial strains and Plasmids used in this study.

| Name | Description | Reference |
| --- | --- | --- |
| *C. crescentus* |  |  |
| *NA1000* | NA1000 is a wild type strain laboratory strain derived from CB15. It lacks holdfast expression due to a mutation in *hfsA*. | (1) |
| *hfsA^+^* | Wild type *hfsA* gene was restored for this strain. | this work |
| Δ*flgDE* (with holdfast) | Clean deletion of flagellar hook *flgD* and *flgE* genes, Filament-less flagellum. Non-motile. | this work |
| Δ*flgDE* | Clean deletion of flagellar hook *DflgD* and *flgE* genes. Filament-less flagellum. Non-motile. | this work |
| Δ*flgDE* Δ*pilA*  (with holdfast) | Clean deletion of flagellar hook *flgD* and *flgE* genes, and *pilA* gene. Filament-less flagellum. Non-motile. Pili negative strain. | this work |
| Δ*pilA* | Clean deletion of the *pilA* gene. Pili negative strain. | (2) |
| Δ*creS*  (with holdfast) | Clean deletion of the *creS* gene. Strain with rod-like cell shape instead of the typical curvature. | this work |
| Δ*motB*  (with holdfast) | Encoding *motB_D33N* variant at the native locus. Stator without proton conductance. Non-motile flagellum. | (3) |
| Δ*motB* | Encoding *motB_D33N* variant at the native locus. Stator without proton conductance. Non-motile flagellum. | this work |
| Δ*dgcB*  (with holdfast) | Encoding *dgcB_E216Q* variant at the native locus. The cyclase is catalytically inactive. | (3) |
| Δ*dgcB* | Encoding *dgcB_E216Q* variant at the native locus. The cyclase is catalytically inactive. | this work |
| Δ*cleA-E* | Clear in frame deletion of *cc0440*, *cc1364*, *cc2249*, *cc3100* and *cc3155*. | (2) |
| *hfsA^-^* Δ*dgcA ΔdgcB* Δ*pleD* Δ*pdeA* Δ*cc0091* Δ*cc0655* Δ*cc0740 Δcc0857* Δ*cc0896* Δ*cc1086* Δ*cc3094* Δ*cc3148* *P_lac_-dgcZ* | rcdG^0^ strain. Clean deletions of all endogenous GGDEF and EAL-domain encoding genes in NA1000. Chromosomal integration of *E. coli* *dgcZ*-3xflag gene under the control of the Plac promoter. | (4) |
| *hfsA^-^* Δ*dgcA ΔdgcB* Δ*pleD* Δ*pdeA* Δ*cc0091* Δ*cc0655* Δ*cc0740 Δcc0857* Δ*cc0896* Δ*cc1086* Δ*cc3094* Δ*cc3148* *P_lac_-dgcZ*  (with holdfast) | SoA1587 strain with restored *hfsA* gene. | (3) |
| Δ*dgcA ΔdgcB* Δ*pleD* Δ*pdeA* Δ*cc0091* Δ*cc0655* Δ*cc0740 Δcc0857* Δ*cc0896* Δ*cc1086* Δ*cc3094* Δ*cc3148* *P_lac_-dgcZ* Δ*hfsK*  (with holdfast) | UJ7771 with clean deletion of gene *hfsK* (*cc3689*), which lowers cohesion of the holdfast. | this work |
| *pilA*^T36C^ | Encoding *pilA_T36C* variant at the native locus. The protein can be labelled with maleimide-reactive dye. | (5) |
| *pilA*^T36C^ Δ*cpaE* | YB8288 with clean deletion of *cpaE* gene. Pili negative strain. | this work |
| *pilA*^T36C^ *rcdG^0^::P_lac_-dgcZ* | SoA1587 strain encoding *pilA_T36C* variant at the native locus. | this work |

| ID | Plasmids | Description | Reference |
| --- | --- | --- | --- |
| pMS100 | pNPTS139 PilA^T36C^ | Used to introduce *pilA_T36C* variant at the native locus. | this work |
| pSA223 | pNPTS138 - xylR_up_-T7-term- *P_lac_-dgcZ-*3xflag | Used for introducing exogenous cyclase *dgcZ* into the genome. | (4) |

Ref 1 = Ref 44 in proof

Ref 2 = Ref 50 in proof

Ref 3 = Ref 28 in proof

Ref 4 = Ref 32 in proof

Ref 5 = Ref 27 in proof

1. Marks ME, Castro-Rojas CM, Teiling C, Du L, Kapatral V, Walunas TL, Crosson S. 2010. The genetic basis of laboratory adaptation in Caulobacter crescentus. J Bacteriol 192:3678–88.

2. Nesper J, Hug I, Kato S, Hee C-S, Habazettl JM, Manfredi P, Grzesiek S, Schirmer T, Emonet T, Jenal U. 2017. Cyclic di-GMP differentially tunes a bacterial flagellar motor through a novel class of CheY-like regulators. Elife 6:e28842.

3. Hug I, Deshpande S, Sprecher KS, Pfohl T, Jenal U. 2017. Second messenger–mediated tactile response by a bacterial rotary motor. Science (80- ) 358:531–534.

4. Abel S, Bucher T, Nicollier M, Hug I, Kaever V, Abel Zur Wiesch P, Jenal U. 2013. Bi-modal distribution of the second messenger c-di-GMP controls cell fate and asymmetry during the caulobacter cell cycle. PLoS Genet 9:e1003744.

5. Ellison CK, Kan J, Dillard RS, Kysela DT, Ducret A, Berne C, Hampton CM, Ke Z, Wright ER, Biais N, Dalia AB, Brun Y V. 2017. Obstruction of pilus retraction stimulates bacterial surface sensing. Science 358:535–538.
